# Integration of machine learning and genome‐wide association study to explore the genomic prediction accuracy of agronomic trait in oats (*Avena sativa* L.)

**DOI:** 10.1002/tpg2.20549

**Published:** 2025-01-08

**Authors:** Jinghan Peng, Xiong Lei, Tianqi Liu, Yi Xiong, Jiqiang Wu, Yanli Xiong, Minghong You, Junming Zhao, Jian Zhang, Xiao Ma

**Affiliations:** ^1^ College of Grassland Science and Technology Sichuan Agricultural University Chengdu China; ^2^ Sichuan Academy of Grassland Science Chengdu China; ^3^ Sichuan Provincial Research Center for Forestry and Grassland Development Chengdu China

## Abstract

Machine learning (ML) has garnered significant attention for its potential to enhance the accuracy of genomic predictions (GPs) in various economic crops with the use of complete genomic information. Genome‐wide association studies (GWAS) are widely used to pinpoint trait‐related causal variant loci in genomes. However, the simultaneous integration of both methods for crop genome prediction necessitates further research. In this study, we integrated ML and GWAS to assess the efficiency of GP for seven key agronomic traits in 195 oat (*Avena sativa*) cultivars from major oat‐growing regions around the world. A total of 94 trait‐associated single nucleotide polymorphisms were identified through the GWAS study. GP studies were conducted using the classical model genomic best linear unbiased prediction (GBLUP) and six ML models. GBLUP performed poorly in predicting all traits except flag leaf width, while none of the ML models consistently provided the best prediction accuracy across all traits. The prediction accuracy of the GWAS‐derived markers was better than that of the use of genome‐wide markers, and plant height had the highest prediction rate at 100 GWAS‐derived markers, and the rest of the traits for which more markers were required. These results play an important role in advancing the use of GP in small oat breeding programs by optimizing the prediction rate of GP and reducing the number of markers, confirming that high prediction rates can be achieved with smaller datasets.

## Core Ideas

- Machine learning often outperformed the prediction accuracy of genomic best linear unbiased prediction.
- Machine learning for matching traits can effectively improve the accuracy of genomic prediction.
- The use of genome‐wide association study‐derived markers improves the accuracy of genomic predictions.

**Abbreviations**
CL: cob length
CV: cross‐validation
FLL: flag leaf length
FLW: flag leaf width
GBLUP: genomic best linear unbiased prediction
GP: genomic prediction
GWAS: genome‐wide association studies
KRR: kernel ridge regression
LD: linkage disequilibrium
MAE: mean absolute error
MAS: marker‐assisted selection
ML: machine learning
MSE: mean square error
PCA: principal component analysis
PH: plant height
QC: quality control
SD: stem diameter
SPP: spikelets per panicle
SVR: support vector regression
TAS: trait‐associated SNPs
TN: tiller number

## 1 INTRODUCTION

Oats (*Avena sativa* L.) are one of the agriculturally significant annual cereals and feed crops, ranking as the sixth most important cereal crop globally (Yan, Zhang, et al., 2023). Oats have gained increasing attention in recent years due to their richness in bioactive components such as soluble β‐glucans, vitamins, polyphenols, and flavonoids, which significantly contribute to the prevention of chronic diseases (Rostamabadi et al., 2022). Additionally, oats are also widely cultivated as pasture grasses, serving as a major source of high‐quality forage for livestock worldwide and playing an important role in soil salinization and desertification management (Baum, 1977; Valentine, 2005). Compared to other crops, oat cultivation requires fewer insecticides, fungicides, and fertilizer treatments and also maintains a lower carbon footprint (Kamal et al., 2022). However, the application of genomics in oat breeding is still weak due to the complexity of its genome and the presence of a large number of repetitive sequences as a result of reticulated heterozygous polyploidy (Peng et al., 2022; Yan et al., 2016). Genome‐assisted breeding offers a shorter breeding cycle than phenotypic selection and can more rapidly alter allele frequencies (Arguello‐Blanco & Sneller, 2023). In the wake of significant advances in high‐throughput sequencing technology and the publication of high‐quality reference genomes, the emergence of genomics‐assisted breeding tools that can be used for oat breeding improvement has facilitated the development of precision breeding in oats.

Genomic prediction (GP or genomic selection) is a principal method of genome‐assisted breeding that helps to accelerate crop breeding and thus increases genetic gain (Crossa et al., 2017; Varshney et al., 2005). Compared with marker‐assisted selection (MAS), GP relies on extensive marker information across the genome and is not confined to markers of known function (Zhou et al., 2022). Agronomic traits that breeders target are often controlled by multiple small effect loci, and while genetic hitchhiking or drift can result in the loss of favorable alleles and reduced selection pressure on the remaining loci, GP provides a way of exploiting small effect QTLs, especially for complex traits with low heritability (Crossa et al., 2017; Varshney et al., 2021). GP is currently becoming a promising tool to improve the efficiency of plant breeding due to the continuous advances in high‐throughput genotyping techniques and the increased accuracy enabled by artificial intelligence (Alemu et al., 2024; Varshney et al., 2021). Machine learning (ML) is probably one of the most widely used branches of artificial intelligence today. Compared to many other methods used in GP, ML methods are able to deal effectively with the high‐dimensional nature of genotypic and phenotypic data and have significant advantages in modeling nonlinear relationships between response and predictor variables, as well as complex interactions between predictor variables (Lourenço et al., 2024), and the introduction of ML methods can lead to better generalization and robustness of GP models (Chafai et al., 2023; Lourenço et al., 2024). Currently, supervised ML methods have been successfully used to predict genomic breeding values (Heslot et al., 2012). In several studies, ML methods such as ridge regression and lasso regression using bridge regularization, support vector regression (SVR), and kernel ridge regression (KRR) outperform classical methods of GP such as genomic best linear unbiased prediction (GBLUP) (Montesinos‐López et al., 2019; Ogutu et al., 2011, 2012). Ornella et al. (2014) reported GP performance studies for different traits in wheat and maize and found reproducing kernel Hilbert spaces and random forest regression to be the best. González‐Camacho et al. (2018) found that support vector machine outperformed classical models in GP of rust resistance in wheat.

Genome‐wide association studies (GWAS) test hundreds of thousands of genetic variants in the genome to identify markers associated with specific traits (Uffelmann et al., 2021). Using GWAS, relevant candidate single nucleotide polymorphism (SNP) markers have been identified for key traits in oats, such as morphological traits hulled and hull‐less, heading date, and seed weight and size (Canales et al., 2021; Peng et al., 2022; Yan, Deng, et al., 2023). Furthermore, GWAS has strong statistical power to estimate marker effects associated with traits, in addition to accurately locating markers corresponding to specific phenotypes. Previous studies integrating GWAS with GP models have demonstrated improved prediction accuracy across crops like tomato (*Solanum lycopersicum* L.), alfalfa (*Medicago sativa* L.), and rice (*Oryza sativa* L.), particularly for complex traits (Anilkumar et al., 2023; Yeon et al., 2024; Zhang et al., 2023). For instance, Anilkumar et al. (2023) found that the inclusion of GWAS‐derived markers significantly enhanced GP for seed rice grain traits. These studies highlight the potential of using GWAS‐derived markers in optimizing the efficiency of GP. However, to date, there is a paucity of studies combining these methods in oats. Developing statistical ML models and optimizing training populations are two main areas actively explored in plant GP research (Alemu et al., 2024). However, studies on improving the efficiency of GP prediction in oats using ML have not been reported. In this paper, we integrated ML and GWAS approaches to assess the efficiency of genomic prediction of agronomic traits in oat populations from around the world. The objectives of this experiment were to: (i) identify genetically related markers for seven agronomic traits of oat germplasm collection using the GWAS, (ii) evaluate the accuracy of GP of GBLUP and different ML models and select the most efficient GP model for each agronomic trait, and (iii) GP using GWAS‐derived markers to calculate trait prediction accuracy and determine the optimal number of markers.

## 2 MATERIALS AND METHODS

### 2.1 Plant materials and field trials

A total of 195 oat germplasms from different regions around the world were obtained from the National Germplasm Repository of the United States (USDA) (Table S1). Sources of the germplasm accessions included 32 countries, such as China, the United States, Turkey, Sweden, the UK, Russia, Finland, France, Germany, Canada, Japan, Australia, and others across Europe, Asia, the Americas, and Africa. All oat accessions were planted in Hongyuan, Sichuan, China (elevation 3504 m, 103.71°E, 30.45°E) and Chengdu, Sichuan, China (elevation 500 m, 102.32°E, 32.46°E) during planting seasons 2021–2022 and 2022–2023, respectively. In each experimental site, all accessions were sown in an incomplete block design with multiple individual plants in a single row. It was repeated three times. Each row was 10 m long, with 0.6 m between rows, and approximately 15–20 plants were grown per row, and site management followed local best practices. and site management followed local best practices.

**Core Ideas**

- Machine learning often outperformed the prediction accuracy of genomic best linear unbiased prediction.
- Machine learning for matching traits can effectively improve the accuracy of genomic prediction.
- The use of genome‐wide association study‐derived markers improves the accuracy of genomic predictions.

### 2.2 Evaluation and statistical analysis of seven agronomic traits

Seven agronomic traits (plant height [PH], cob length [CL], flag leaf length [FLL], flag leaf width [FLW], spikelets per panicle [SPP], stem diameter [SD], and tiller number [TN]) were evaluated. Five individuals were randomly harvested from each row at the milk stage. PH and CL were measured on the main spike, and FLL and FLW were measured at the longest and widest positions on the flag leaf, and SD was measured at the thickest part of the main spike stalk. SPP was determined by counting the number of fertile spikelets in the main spike. FN was determined by counting the number of all the main stems and lateral branches.

The R package lme4 (Bates et al., 2015) was used to analyze genotype‐by‐environment (G × E) interactions and population‐by‐phenotype variance to calculate the best linear unbiased prediction (BLUP) using the following model:

$$y_{ijk}=\mu +G_{i}+\beta_{j}+\alpha_{k}+{\left(G\times \beta \right)}_{ij}+e_{ijk}$$

where $y_{ijk}$ is the phenotype of genotype *i* from group *k* in environment *j*, *μ* is the general mean, $G_{i}$ is the genotype effects of the *i*th accession, $\beta_{j}$ is the effect of the *j*th environment, $\alpha_{k}$ is the effect of group *k* with *k* ∈ {groups 1 and 2}, ${\left(G\;\times \;\beta \right)}_{ij}$ is genotype by environment interaction effect of genotype *i* from group *k* in environment *j* with ${\left(G\;\times \;\beta \right)}_{ij}\;∼\;N\left(0,\sigma_{{G\;\times \;\beta )}_{k}}^{2}\right)$ independent, *e_ijk_* is the error of individual *i*, group *k* in environment *j* with $e_{ijk}\;∼\;N\left(0,\sigma_{E}^{2}\right)$ independent and identically distributed normal distribution. All random effects are assumed to be independent of each other.

### 2.3 DNA extraction, super‐GBS sequencing, and SNP calling

For each oat germplasm resource, five vigorous plants having 0.5 g of young leaf samples were randomly selected at the seedling stage and collected in 2 mL centrifuge tubes. They were immediately frozen in liquid nitrogen and stored at −80°C until used for DNA extraction. Genomic DNA was extracted using a modified CTAB protocol method, as previously described. The mass and concentration of DNA were determined using a NanoDrop 2000 spectrophotometer (Thermo Fisher Scientific), and a working solution was prepared with a concentration of 30 ng/µL. Sequencing was performed using Super‐GBS. The DNA was digested by PstI‐HF/MspI. Subsequently, the size of the recovered fragments was adjusted by modifying the volume ratio of the magnetic bead solution to the ligated product using a modified magnetic bead recovery system. Finally, the fragments were amplified by polymerase chain reaction using high‐fidelity enzymes to construct hybrid libraries, and the sequencing of each pooled library was performed on the Illumina Hiseq PE150 platform.

The FastQC software (Andrews, S., 2010) was used to perform quality control (QC) on the raw reads from the multi‐sample mixing pool. Process_radtags from the stacks package was used to remove reads containing splice sequences from the mixing pool raw reads and split them into single‐sample raw reads based on the correspondence between the library samples and the barcode. The fastx_trimmer, from the fastx toolkit package, was used to remove enzymatic cleavage site sequences and all bases with a QC score of less than 20 at the 3′ end. Using the fastx_trimmer program in the fastx toolkit package, the clean reads were obtained by removing all the bases with a fastqc QC score of less than 20 at the 3′ end of the digest site and all the bases with a fastqc QC score of less than 20 at the 3′ end of the digest site. Clean Reads were aligned to the oat reference genome cv sang_V1.1 using bowtie2 software (Langmead & Salzberg, 2012). SNP detection was carried out using GATK software (McKenna et al., 2010). A total of 77,835 SNP markers were obtained using VCFtools (Danecek et al., 2011) after screening for insertion deletion (Indel), minor allele frequency (>1%), heterozygosity (<20%), and percentage of missing values (<70%). Finally, Beagle (Browning & Browning, 2009) (V4.1) was used to populate the filtered SNP with default parameters.

### 2.4 Population analysis and heritability estimation

Population structure analyses were performed on two to eight genetic populations K using Admixture software (Alexander et al., 2009), with linkage disequilibrium (LD) filtering of variation to improve the accuracy and interpretability of the analyses. Principal component analysis (PCA) was performed using PLINK (Slifer, 2018) (v1.9). Phylogenetic trees were constructed using the iqtree (Nguyen et al., 2015) maximum likelihood method. Weir and Cockerham's fixation index (Fst) was calculated by VCFtools (Danecek et al., 2011) (v0.1.16) for pairwise subpopulations on a per SNP basis and sliding window (30‐kb window) separately.

The LDAK‐Thin (Gazal et al., 2019) model was used to calculate the heritability of seven complex traits in oats. This model can summarize statistics and highly complex genetic models from GWAS. Estimates of heritability for complex traits are more accurately assessed based on the overall contribution of genes to the phenotype. The LDAK‐thin model first sparsifies the variants using the parameters “–window‐prune 0.98 and –window‐kb 100” to ensure that no two SNPs have an *r* ^2^ value between them greater than 0.98. It then calculates the kinship matrix that needs to be introduced into the computation and introduces the first four principal components of the PCA as covariates. The following model was used for the heritability calculation:

$$E\left[h^{2}|j\right]=w_{j}\times p_{j}^{0.75}\times \tau_{1}+\sum \left(b_{jk}\times \tau_{k}\right)$$

where $E[h^{2}|j]$ denotes the expected value of heritability for a given SNP*j*. Heritability here refers to the degree of contribution of SNP*j* to the phenotypic variation. $w_{j}$ denotes the weight of SNP*j*. *p_j_* denotes the marginal allele frequency (MAF) of SNP*j*. $\tau_{1}$ denotes the baseline heritability, which is a constant used to adjust the baseline magnitude of the heritability. $\sum (b_{jk}\times \tau_{k})$ indicates the effect of other functional indicators on the heritability of SNP*j*, where $b_{jk}$ the strength of the effect of the functional indicator on SNP*j*, and $\tau_{k}$ is the effect size.

### 2.5 Analysis of genome‐wide association study

GWAS were performed on the phenotypes estimated in the four environments as well as their calculated BLUP values. The analysis utilized the GAPIT3 (J. Wang & Zhang, 2021) package in R, applying four different models: (1) The efficient mixed model association eXpedited, (2) Bayesian information linkage disequilibrium iteratively nested keyway, (3) fixed and random model circulating probability unification, and (4) settlement of MLM under progressively exclusive relationship. Multiple test corrections were applied to key adjusted *p*‐values to assess the significance of associations, according to the Bonferroni criterion (*p* = 0.05/*n*, where *n* is the number of SNP markers used in GWAS). The Manhattan and *Q*–*Q* plots of the GWAS results were generated using the CMplot package in R (Yin et al., 2021).

### 2.6 Genomic prediction models

GBLUP and six ML methods were employed for GP. In this study, a grid search approach was utilized, along with an internal fivefold cross‐validation (CV) strategy, to tune the hyperparameters.

The GBLUP model is a mixed model containing both fixed and random effects, and the model equation is as follows:

y=Xβ+Zu+e

where **y** is the vector of observed phenotypes, **X** is the design matrix for the fixed effects, **β** is the vector of fixed effects, **Z** is the design matrix for the random genetic effects, **u** is the vector of random genetic effects, assumed to be normally distributed as $u\;∼\;N(0,G\sigma_{u}^{2})$, **G** is the genomic relationship matrix calculated from SNP markers, representing the covariance between the genetic values of the individuals, **e** is the vector of residual errors, with $e\;∼\;N(0,I\sigma_{e}^{2})$, **I** is an identity matrix, and $\sigma_{u}^{2}$ and $\sigma_{e}^{2}$ are the variances of the genetic and residual errors, respectively.

Ridge regression, minimal absolute contraction and selection operators, and elastic nets are special cases of bridge regression, and the generalized bridge regression method is:

$${\hat{\beta }}_{\mathrm{bridge}}=\mathrm{minimize}\sum_{i=1}^{n}{\left(y_{i}+\sum_{j=1}^{p}\;x_{ij}\beta_{j}\right)}^{2}+\lambda \sum_{j=1}^{p}\beta_{j}^{\gamma }$$

where *y_i_* is the phenotypic value of the *i*th sample, *X_ij_* is the value of the *i*th sample at the *j*th locus, *β_j_* is the effect size at the *j*th locus, and *λ* is a parameter controlling the strength of regularization. Bridge regression automatically selects relevant predictors at 0 < *γ* ≤ 1, shrinks the coefficients at *γ* > 1, and reduces to subset selection at *γ* = 0. The bridge estimate reduces to LASSO estimation when *γ* = 1 and to ridge estimation when *γ* = 2.

Specifically, ridge regression deals with the problem of covariance by adding an L2 regular term and is suitable for situations where there is a high degree of correlation between features. In genotype data analysis, there may be high correlation between features (i.e., gene loci), and ridge regression prevents overfitting and improves the generalization ability of the model, and the ridge regression method is:

$${\hat{\beta }}_{\mathrm{Ridge}}=\;\mathrm{minimize}\sum_{i=1}^{n}{\left(y_{i}+\sum_{j=1}^{p}x_{ij}\beta_{j}\right)}^{2}+\lambda \sum_{j=1}^{p}\beta_{j}^{2}$$

KRR permitted regularized linear regression under nonlinear transformations of the input features by introducing a kernel function. The ElasticNet regression method is:

$${\hat{\beta }}_{\mathrm{KRR}}=\;\mathrm{minimize}\beta \sum_{i=1}^{n}{\left(y_{i}\;-\;K\left(x_{i},\;X\right)\beta \right)}^{2}+\lambda ∥\beta {∥}^{2}$$

where $K(x_{i},\;X)$ is obtained from the kernel function, which represents the nonlinear similarity between the input data *x_i_* and the training set *X*, and λ is the regularization parameter.

Lasso regression promotes sparse solutions by adding an L1 regular term that allows for automatic feature selection, compressing the coefficients of unimportant loci to 0. This is useful for working with genomic data that have a large number of features, but only a few of which are important, and the Lasso regression method is:

$${\hat{\beta }}_{\mathrm{Lasso}}=\;\mathrm{minimize}\sum_{i=1}^{n}{\left(y_{i}+\sum_{j=1}^{p}x_{ij}\beta_{j}\right)}^{2}+\lambda \sum_{j=1}^{p}\left|\beta_{j}\right|$$

ElasticNet combines the advantages of Lasso and ridge regression to handle both high correlation between features and feature selection by adding both L1 and L2 regular terms. This makes ElasticNet well suited for genomic data analysis, especially when there are a large number of interrelated features in the dataset. The ElasticNet regression method is:

$$\begin{array}{c} {\hat{\beta }}_{\mathrm{ElasticNet}}=\;\mathrm{minimize}\sum_{i=1}^{n}{\left(y_{i}+\sum_{j=1}^{p}x_{ij}\beta_{j}\right)}^{2}+\lambda \sum_{j=1}^{p}\left|\beta_{j}\right|+\lambda \sum_{j=1}^{p}\beta_{j}^{2} \end{array}$$

SVR is used to deal with quantitative responses, using linear or polynomial kernel functions to map the input space (labeled dataset) to a high‐dimensional feature space for predicting complex traits.

Linear SVR is the most basic form and is applicable when there is a linear relationship between genotype and phenotype. The model of linear SVR can be expressed as:

$$y_{i}=\beta_{0}+x_{i}^{t}\beta +\varepsilon_{i}$$

where $y_{i}$ represents the phenotype, **x** *_i_* is the vector of genotypic markers for the *i* individual, β is the vector of coefficients, $\beta_{0}$ is the intercept, and *ϵ_i_* is the error term. The optimization problem for SVR‐linear is defined as:

$$\min\beta_{0},\;\beta (\frac{1}{2}\beta^{2}+C\sum_{i=1}^{n}\max\left(0,\left|\beta_{0}-X_{i}^{t}\beta \right|-\varepsilon \right)$$

The polynomial SVR model extends the linear model by incorporating a polynomial kernel, allowing it to capture nonlinear relationships. The kernel function used is defined as:

$$K\left(x_{i},x_{j}\right)={\left(\gamma \langle x_{i},x_{j}\rangle +r\right)}^{d}$$

where $\left\langle x_{i},x_{j}\right\rangle$ is the dot product between the genotypic marker vectors of the *i* and *j* individuals, γ is the scale factor, r is the independent term coefficient, and *d* is the polynomial degree. The optimization problem for SVR‐poly is similar to SVR‐linear, with the replacement of the linear term $X_{i}^{t}\beta$ by the kernelized feature mapping. In this study, the polynomial degree *d* and the parameters *C*, γ, and *ϵ* are tuned by a grid search.

For all GP models, accuracy was measured by the Pearson correlation between *yc* (corrected phenotype) and *yp* (predicted value). Accuracy of GPs was estimated using a fivefold CV, with training and testing populations randomized into 80% and 20% genotypes, respectively. Prediction unbiasedness was calculated by regressing observed values (*yc*) against predicted values (*yp*) in the validation population. The fivefold CV procedure was repeated 500 times, and the final prediction accuracy was determined as the mean of these replications. Additionally, mean square error (MSE) and mean absolute error (MAE) were employed to evaluate the performance of the regression models in this study (Alves et al., 2021). MSE accounts for both prediction accuracy and bias; a smaller MSE value indicates greater model accuracy in describing experimental data. MAE more accurately reflects the actual errors in predicted values. The formulas for MSE and MAE are as follows:

$$\mathrm{MSE}=\frac{1}{\omega }\;\sum_{i=1}^{\omega }{\left(f_{i}-y_{i}\right)}^{2}$$

$$\mathrm{MAE}=\frac{1}{\omega }\;\sum_{i=1}^{\omega }\left|f_{i}-y_{i}\right|$$

where ω denotes the number of phenotypic traits for each CV test multiple in the fivefold CV, **f** is a vector of predicted values (*yp*), and **y** is a vector of observed values (*yc*). The final MSE and MAE are the mean of 500 replicate determinations.

## 3 RESULTS

### 3.1 Population characterization

**FIGURE 1:**
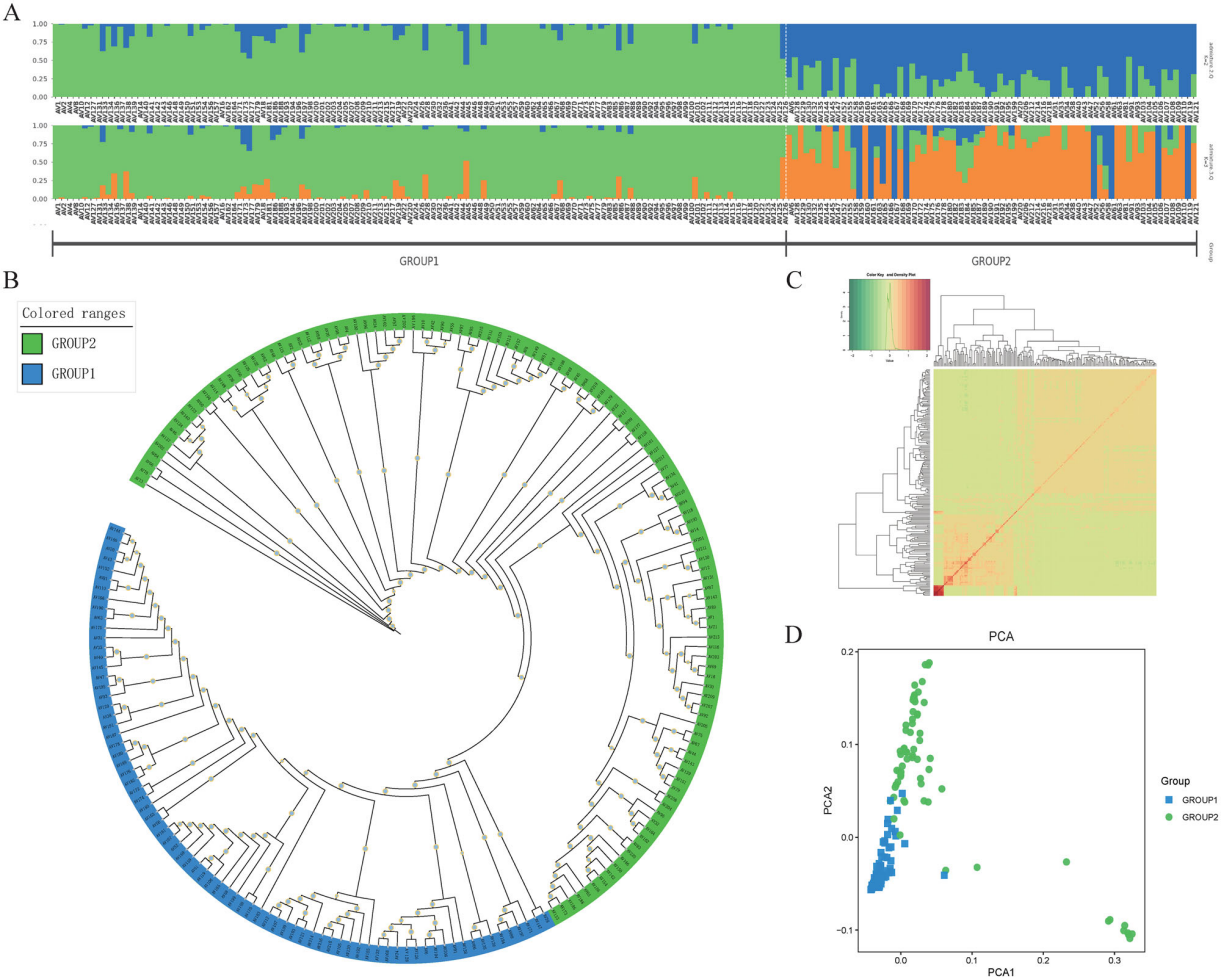
Population structure of 195 oats. (A) Population structure (*K* = 2 and 3) with each vertical line representing one oat germplasm and different colors representing different clusters and subgroups, (B) phylogenetic tree of 195 oats, (C) heatmap of kinship matrices, and (D) principal component analysis (PCA) plot.

The genetic relationships among the 195 oat germplasms were shown in Figure 1. Phylogenetic trees revealed that the 195 oats could be categorized into two main groups (Figure 1B), with 75 germplasms in Group 1 and 120 in Group 2. Investigations of population structure using Admixture at different K‐means levels also predicted (Figure S1) that the optimal number of subpopulations is approximately *K* = 2 (Figure 1A). Additionally, we performed PCA to better resolve the population structure of the two populations (Figure 1D), where PC1 and PC2 accounted for 24.57% and 9.68% of the variance, respectively. The population structure of Group 1 was compact, whereas that of Group 2 was more dispersed. The Weir and Cockerham fixation index (Fst) value of 0.24 was calculated for the two groups, indicating a significant differentiation between the two groups. Similarly, the kinship matrix (Figure 1C) clearly delineated these materials into two distinct groups. Overall, these results support the genetic diversity results and suggest a stable boundary and clear genetic background between the two groups.

### 3.2 Phenotypic variations and heritability estimation of seven agronomic traits

**FIGURE 2:**
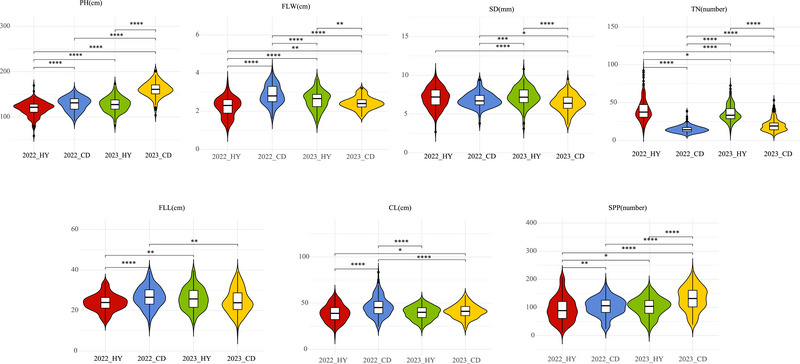
Phenotypes of the seven agronomic traits in four environments. PH represent plant height; SD represents stem diameter; FLL and FLW represent flag leaf length and flag leaf width, respectively; CL represents cob length; TN represents tiller number; SPP represents spikelets per panicle. *p < 0.05, **p < 0.01, ***p < 0.001, and ****p  <  0.0001.

The seven agronomic traits of oat, TN, FLL, FLW, CL, SD, SPP, and PH, were measured in the two different environments in 2022 and 2023, respectively. The means, maxima, minima, variances, and coefficients of variation (CV) were counted (Table S2). The CVs of the seven agronomic traits in oats ranged from 10% to 48%, with large variances, with larger coefficients of variation for the TN and SPP traits. Phenotypic differences across all traits were significant and closely approximated by a normal distribution (Figure S2). And the performance of the seven agronomic traits was further analyzed in different environments, and, as expected, there were differences in the performance of agronomic traits in different environments (Figure 2). Consequently, we integrated data on environment, genotype, and genotype subgroup differentiation to compute BLUP values, thus minimizing bias from genotype–environment interactions. The heritability that can be explained by SNPs was calculated using the LADK‐Thin model using BLUP values combined with the 77,835 SNP markers data (Table S3). All evaluated traits exhibited moderate to high heritability, ranging from 0.55 to 0.76, with SPP showing the lowest and SD the highest heritability.

### 3.3 Genome association mapping

**FIGURE 3:**
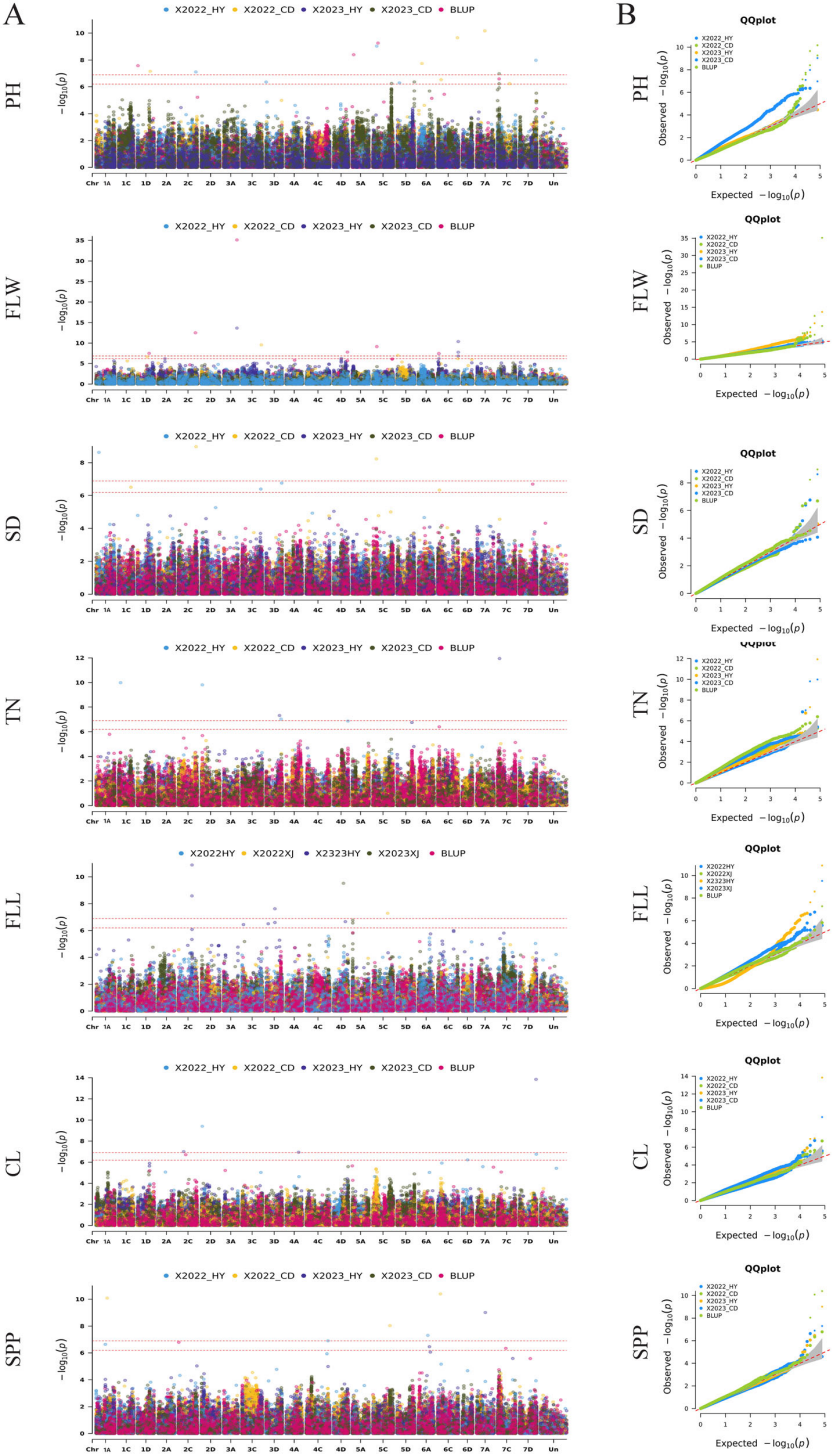
Genome‐wide association studies (GWAS) analysis of seven oat agronomic traits related to single nucleotide polymorphism (SNP), with the color and shade of the dots referring to different environments as well as the strength of the association. A quantile–quantile (*Q*–*Q*) plot of seven agronomic traits. B quantile–quantile plot of seven agronomic traits. The two red horizontal dashed lines indicate the genome‐wide significance thresholds for 1% and 5% Bonferroni corrections, respectively. BLUP, best linear unbiased prediction; CL, cob length; FLL, flag leaf length; FLW, flag leaf width; PH, plant height; SD, stem diameter; SPP, spikelets per panicle; TN, tiller number.

To identify SNP markers associated with seven agronomic traits, we analyzed genotypic and phenotypic data using four models in the R software GAPIT3 (J. Wang & Zhang, 2021) selecting the optimal results as the final association outcomes (Figure 3, Table S4). GWAS analyses were conducted for each of the four phenotypes across various environments and BLUP values. In this study, a total of 94 trait‐associated SNPs (TASs) were identified after applying Bonferroni correction at a 5% significance threshold (Table S5). Among them, three TASs were significant in at least two environments: two TASs, chr3A_378078108 and chr6C_25741715, associated with FLW, were located on chromosomes 3A and 6C, respectively, and one TASs, chr5A_16429536, associated with FLL, was located on chromosome 5A. In addition, there was also one TASs that was present in both SPP and FLL were both identified.

### 3.4 Prediction accuracy in cross‐validation comparison of ML method with GBLUP

**FIGURE 4:**
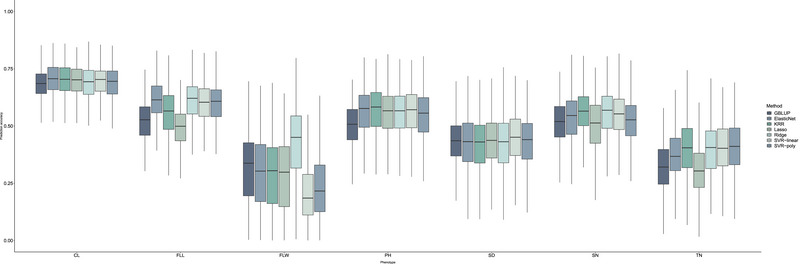
Accuracy of seven different genomic prediction methods for seven agronomic traits in oats. CL, cob length; FLL, flag leaf length; FLW, flag leaf width; GBLUP, genomic best linear unbiased prediction; KRR, kernel ridge regression; PH, plant height; SD, stem diameter; SPP, spikelets per panicle; SVR, support vector regression; TN, tiller number.

Prediction of seven agronomic traits based on all markers was performed using seven models (Figure 4), including the GBLUP model and six ML models: Lasso, Ridge, ElasticNet, KRR, SVR‐linear, and SVR‐poly. Results were validated through fivefold CV. Among the ML models, the Ridge model achieved the highest prediction accuracies for FLL and FLW traits at 60.866% and 41.682%, respectively, while the SVR‐linear model was most accurate for SPP and CL traits, with accuracies of 68.7% and 44.8%. The Lasso model demonstrated the poorest performance across FLL, TN, SPP, PH, and SD traits. However, it performed best in predicting SD, with an accuracy of 46.961%. The SVR‐linear model and the KRR model excelled in predicting PH and CL, respectively. Additionally, MSE and MAE are utilized for evaluating model performance (Table S6). Significant variations in MAE and MSE were observed among different traits, likely attributable to the diverse genetic backgrounds of these agronomic traits. ML methods outperformed GBLUP in terms of both MSE and MAE. Among the ML methods, Ridge achieved the best results for FLL and FLW, with MSEs of 0.008 and 0.112 and MAEs of 0.0001 and 0.012, respectively. KRR was most effective for SPP, PH, and CL, posting MSEs of 14.341, 15.162, and 12.965, and MAEs of 205.68, 229.88, and 168.08, respectively. The best performing ML model for TN was SVR‐poly, with an MSE of 17.311 and an MAE of 299.66. For SD, the top performer was SVR‐linear, with an MSE of 0.105 and an MAE of 0.011.

### 3.5 Using GWAS‐derived markers for GP

**FIGURE 5:**
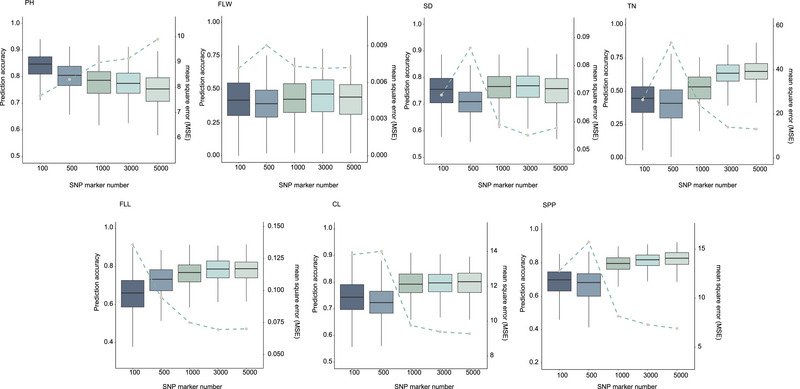
Box plots of the mean of prediction accuracies among genome‐wide association studies (GWAS)‐related markers for seven agronomic traits in oats. CL, cob length; FLL, flag leaf length; FLW, flag leaf width; PH, plant height; SD, stem diameter; SNP, single nucleotide polymorphism; SPP, spikelets per panicle; TN, tiller number.

In order to reduce the number of markers used in GP and to find the optimal number of markers to improve prediction efficiency, we used the model with the highest prediction rate in the model comparison and further predicted the seven agronomic traits using different numbers of TASs (top 100–5000) (Figure 5). The results showed that the prediction mean accuracy of the six traits in showed a decreasing and then increasing trend, except for the PH trait. FLL, FLW, and SD had the best prediction structure at 3000 marker numbers, and the remaining three traits required a larger number of markers (5000). Interestingly, the prediction rate of PH decreased gradually with the increase of marker number and reached the lowest at 5000. And the MSE and MAE also performed consistently with the predicted rates. Meanwhile, the prediction accuracy values using GWAS‐derived markers were higher than those using all markers, except for the FLW and TN traits, where the prediction accuracy using 500 GWAS‐derived markers was lower than the prediction accuracy using all markers.

## 4 DISCUSSION

### 4.1 Potential of GWAS in genetic improvement of oats

GWAS are powerful tools for identifying trait‐related markers in genetic studies. Previous research has successfully used GWAS to identify loci associated with important traits in oats, such as hull‐less (nude) traits and heading date (Canales et al., 2021; Peng et al., 2022). In our study, 94 TASs were identified for seven agronomic traits in oats. Most of the TASs were detected in only one environment, with only three TASs identified across multiple environments. This variability may be attributed to significant environmental differences between study locations and the complex nature of agronomic traits, which are influenced by multiple genetic factors that may only express their effects in specific environments, leading to inconsistencies in the TASs detected across different environments (Rispail et al., 2018). Additionally, a significant TAS was identified in both spikelet per plant (SPP) and FLL. Flag leaves, as major photosynthetic organs, are active during maturation, providing essential energy and material for seed filling (Initiative, 2020). FLL and SPP share biological pathways related to hormone regulation and the partitioning of photosynthetically assimilated material during growth and development. Therefore, this locus may have pleiotropic effects on both FLL and SPP. Nevertheless, the TASs identified by GWAS in our study had limited impact on each of these agronomic traits, likely because these traits are complex and the contribution of individual genes may be minimal, resulting in modest effects from single SNPs. Consequently, it is challenging to use these loci for MAS to comprehensively improve oat agronomic traits (Jannink et al., 2010).

### 4.2 ML method to improve the prediction accuracy of GP

GP can make more comprehensive use of genomic information, especially when dealing with complex traits, and can better capture the cumulative effects of small effector loci to improve the efficiency of selection (Alemu et al., 2024). The use of ML methods to further improve the accuracy of GP is a promising direction that has already yielded better results in rice, maize, and alfalfa crops (Kaler et al., 2022). In this study, we demonstrate the superiority of the ML method by investigating its accuracy in oat genome prediction and comparing it with the classical method GBLUP. ML has potential advantages when dealing with complex genetic systems. In the presence of nonlinear or epistatic relationships, traditional linear models usually assume that the genetic determinants of a trait must follow a specific distribution and are therefore difficult to apply to all traits (Zingaretti et al., 2020). The ML method can better take into account the correlations and interactions between markers, thus improving the modeling of complex genetic systems and enabling them to more accurately capture the relationship between genotype and phenotype. This is important for improving the prediction accuracy of complex traits (Piles et al., 2021). In addition, ML methods have advantages in dealing with nonadditive effects, as they are more flexible in accommodating different types of genetic effects and can predict traits more accurately, especially in the presence of multi‐locus effects (Brault et al., 2022).

Our fivefold CV experiments align with previous studies that demonstrated varying performance of ML models based on traits (Gill et al., 2022; X. Wang et al., 2022). In this study, we integrated GWAS‐derived markers, resulting in improved prediction accuracy for traits such as FLW and plant height, which is the same as that observed in previous studies in crops such as rice and alfalfa, suggesting that combining GWAS‐derived markers to improve GP has potential (Anilkumar et al., 2023; Zhang et al., 2023). However, the performance of the different traits suggests that further improvements are necessary to optimize marker selection for each trait, especially in polyploid species such as oats. In GP studies, it is common to improve the breeding process by modeling the relationship between SNP markers and target traits to predict the performance of new individuals or to understand the contribution of genes to traits. However, usually a large number of SNP marker inputs and fewer observations (trait data of individuals) may pose the challenge of model overfitting (de Los Campos et al., 2015). This phenomenon has led to the emergence of different penalization (regularization) methods (Montesinos‐López et al., 2022). In our study, the lasso model performed the worst in FLL, TN, SPP, and PH traits, while the Elsticnet and ridge models had high prediction rates in all these traits, suggesting that model selection for these traits affect (influence) the prediction accuracy. The lasso, Elsticnet and Ridge regression are the three forms of regularization for the bridge model (Kipp & Warmenhoven, 2022). The lasso model is an L1 regularization model, which tends to produce sparse solutions and ignore the effect of small effect SNPs, leading to the neglect of small effect SNPs in complex traits, whereas the elsticnet (combined L1 and L2 regularization) and the ridge (L2 regularization) models retain more small effect SNPs (Brault et al., 2022). Therefore, for the different traits, it is necessary to find ML methods corresponding to the genetic structure of this trait; otherwise, ML models do not always have a high prediction accuracy.

### 4.3 Improving GP efficiency using GWAS of related markers

In this study, we used GWAS to estimate marker effects associated with traits and investigated GP performance using different numbers of marker sets. We found the following potential advantages of using GWAS‐derived markers for GP using ML methods. (1) The number of useful markers is a key factor in determining the accuracy of GP prediction, and trait‐related markers should be considered in genomic selection for plant breeding, which can result in higher prediction accuracy. According to our results, the average prediction rate of the GWAS‐derived marker set improved the prediction accuracy to varying degrees compared with all genomic markers, which is consistent with previous findings for other crops such as soybean (Glycine max L.), rice, and alfalfa (Anilkumar et al., 2023; Shi et al., 2022; Zhang et al., 2023). (2) Introducing ML methods that can efficiently handle high‐dimensional data has significant advantages in improving GP prediction. However, this usually comes at the cost of an extremely high computational burden (Shen et al., 2024). And as the number of markers increases to millions or even tens of millions, the computational cost becomes increasingly high (Lourenço et al., 2024). Genome‐wide markers may contain a large number of redundant, low information content, while GWAS‐derived markers tend to be highly correlated with the target traits, and GWAS estimation of marker effects offers the possibility of reducing the number of markers to improve computational efficiency. Furthermore, we found that the prediction accuracy of PH was maximal at 100 markers, and the introduction of more markers led to a decrease in prediction accuracy, while the opposite was true for other agronomic traits. This may be due to the fact that traits with relatively simple genetic backgrounds are regulated by a small number of primary effector QTLs, and the introduction of more markers may include many markers that are not related or very weakly associated with the target phenotype, and these markers may introduce additional noise that interferes with the model's identification and weight assignment of the markers that truly affect the phenotype (Chen et al., 2023). In addition, the representativeness of the oat germplasm resources used in this study may limit the use of the model on a larger scale. Although the 195 accessions represent different geographical regions, they still may not fully reflect the global genetic diversity of oats. Under‐representation of specific regions or environments may affect the accuracy and applicability of prediction models. Future studies should consider expanding the genetic diversity of germplasm collections to improve the robustness of GP models in different oat populations.

In conclusion, when using ML approaches for GP, there is a need to screen the best ML models for different traits. GWAS‐derived SNP markers have the potential to improve GP efficiency, but there is a need to find the right number of markers to use for prediction for the trait. The use of GWAS‐derived SNP markers and ML approaches in GP offers a compelling avenue to improve breeding efficiency and accuracy, especially when fast, cost‐effective solutions are required.

## AUTHOR CONTRIBUTIONS

**Jinghan Peng**: Conceptualization; investigation; methodology; visualization; writing—original draft. **Xiong Lei**: Conceptualization; formal analysis; supervision; writing—review and editing. **Tianqi Liu**: Software. **Yi Xiong**: Methodology. **Jiqiang Wu**: Software. **Yanli Xiong**: Software. **Minghong You**: Validation. **Junming Zhao**: Visualization. **Jian Zhang**: Data curation. **Xiao Ma**: Conceptualization; formal analysis; investigation; methodology; project administration; resources; supervision; validation; writing—original draft; writing—review and editing.

## CONFLICT OF INTEREST STATEMENT

The authors declare no conflicts of interest.

## Supporting information



Supplementary Information

## Data Availability

The raw sequence data reported in this article have been deposited into the China National GeneBank DataBase under accession number CNP0005721.
