# Supplementary material for: Integration of machine learning and genome‐wide association study to explore the genomic prediction accuracy of agronomic trait in oats (Avena sativa L.)
Source: Plant Genome. 2025 Jan 8;18(1):e20549. doi: 10.1002/tpg2.20549 (PMC11711298; doi:10.1002/tpg2.20549)
Supplement: Supplementary file 1 — Supplementary Information [file TPG2-18-e20549-s001.pdf]

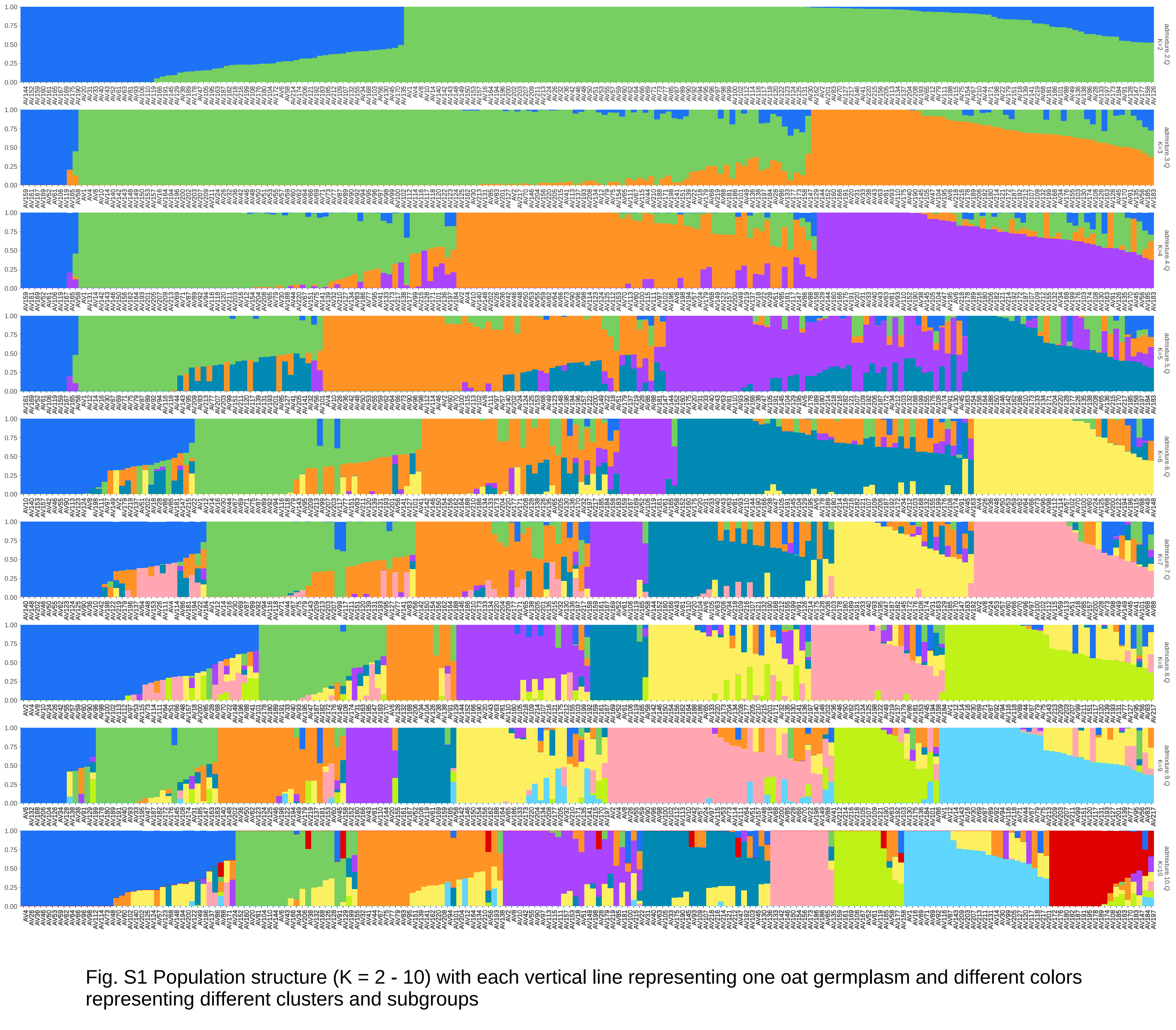

Fig. S1 Population structure (K = 2 - 10) with each vertical line representing one oat germplasm and different colors representing different clusters and subgroups

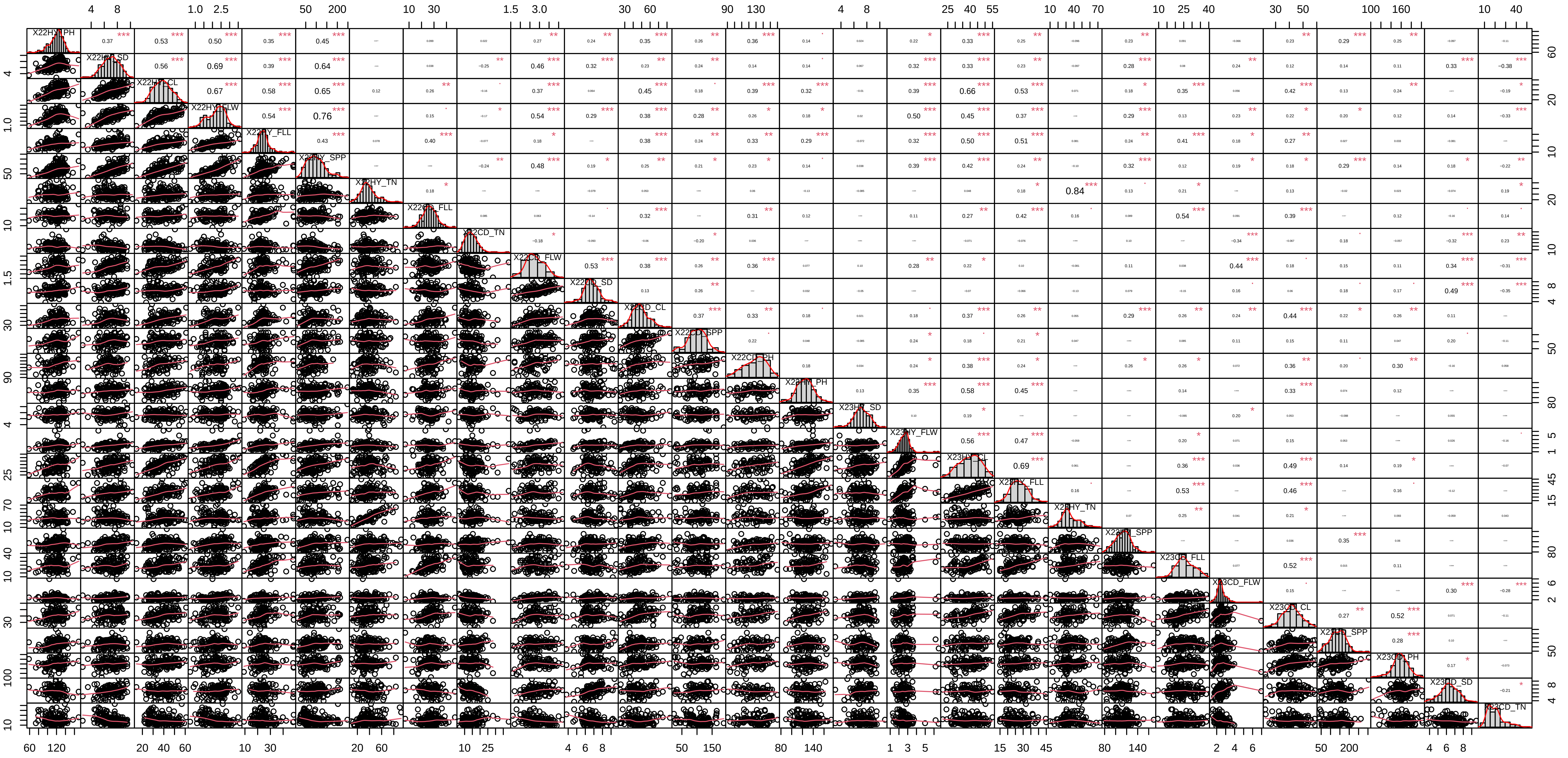

Fig. S2 Scatter plots and correlation coefficients of seven agronomic trait tables for 195 oats in four environments are below and above the diagonal, respectively. PH, represent plant height; SD, represent stem diameter; FLL and FLW represent flag leaf length and flag leaf width,respectively; CL, represent cob length; TN, represent tiller number; SPP, represent spikelets per panicle. *P < 0.05, **P < 0.01, and ***P < 0.001

Table S1. The basic information of 195 oat accessions.

taxa	Accession	Crop	Name	Species	Origin
AV1	447297	Oat	Kuerle	*Avena sativa*	China
AV10	264745	oat	Victory No.1	*Avena sativa*	Japan
AV100	265456	oat	Juha	*Avena sativa*	Finland
AV101	CIav1684	oat	Danish Island	*Avena sativa*	USA
AV102	265459	oat	Tammi	*Avena sativa*	Finland
AV103	CIav1686	oat	White Bonanza	*Avena sativa*	USA
AV104	265961	oat	Stromogul Black	*Avena sativa*	Canada
AV105	CIav1687	oat	Polonea	*Avena sativa*	USA
AV106	266266	oat	WIR 4783/I	*Avena sativa*	Syria
AV107	CIav1692	oat	White Maine	*Avena sativa*	USA
AV108	266273	oat	Sort 4	*Avena sativa*	Serbia
AV109	CIav1693	oat	Japan	*Avena sativa*	USA
AV110	266276	oat	WIR 2590	*Avena sativa*	Georgia
AV111	277050	oat	Weibull's 16004	*Avena sativa*	Sweden
AV112	182493	oat	Magan 044	*Avena sativa*	Georgia
AV113	277052	oat	Weibull's 16229	*Avena sativa*	Sweden
AV114	183607	oat	Otterbacher	*Avena sativa*	Austria
AV115	279449	oat	Max	*Avena sativa*	Denmark
AV116	183844	oat	10735	*Avena sativa*	Turkey
AV117	280431	oat	L'govskij1026	*Avena sativa*	Georgia
AV118	184009	oat	204	*Avena sativa*	Serbia
AV119	287271	oat	AVE 141/62	*Avena sativa*	Greece
AV12	221285	oat	Kyto	*Avena sativa*	Finland
AV120	184010	oat	205	*Avena sativa*	Serbia
AV121	CIav846	oat	O.A.C.No.72	*Avena sativa*	Canada
AV122	CIav1592	oat	Alexander	*Avena sativa*	USA
AV123	CIav1010	oat	Swedish Select	*Avena sativa*	USA
AV124	CIav1593	oat	Lincoln	*Avena sativa*	USA
AV125	CIav1268	oat	Early Pearl	*Avena sativa*	USA
AV126	CIav1594	oat	Wisconsin Pedig	*Avena sativa*	USA
AV127	CIav1327	oat	O.A.C.No.500	*Avena sativa*	Canada
AV128	CIav1595	oat	Black Tartarian	*Avena sativa*	USA
AV129	CIav1454	oat	Victor	*Avena sativa*	United Kingdom
AV130	CIav1596	oat	Black Tartar	*Avena sativa*	United Kingdom
AV131	372075	oat	Stendskij Pozdn	*Avena sativa*	Latvia
AV132	CIav1604	oat	Sparrowbill	*Avena sativa*	USA
AV133	390929	oat	Li-Chia-Ch'ang	*Avena sativa*	China
AV134	CIav1605	oat	White Egyptian	*Avena sativa*	USA
AV135	401771	oat	Auslese Aus Nu	*Avena sativa*	France
AV136	CIav1609	oat	Seizure	*Avena sativa*	USA
AV137	401823	oat	Phatex	*Avena sativa*	Poland
AV138	CIav1618	oat	Setosa	*Avena sativa*	USA
AV139	405734	oat	VI/25	*Avena sativa*	Macedonia
AV14	221291	oat	Osmo	*Avena sativa*	Finland
AV140	CIav1627	oat	Swedish Select	*Avena sativa*	USA
AV141	411425	oat	CAV3431	*Avena sativa*	Turkey
AV142	57277	oat	Clav2051	*Avena sativa*	China

AV143	420634	oat	L'govskij 1026	*Avena sativa*	Georgia
AV144	60768	oat	Tula	*Avena sativa*	Georgia
AV145	436049	oat	186	*Avena sativa*	Chile
AV146	69826	oat	8233	*Avena sativa*	China
AV147	436060	oat	292	*Avena sativa*	Chile
AV148	73882	oat	344-228	*Avena sativa*	China
AV149	436074	oat	336	*Avena sativa*	Chile
AV150	76815	oat	Ling Tang Mai	*Avena sativa*	China
AV151	267080	oat	Artemovskij 1	*Avena sativa*	United Kingdom
AV152	86176	oat	1329	*Avena sativa*	Georgia
AV153	269144	oat	Blenda	*Avena sativa*	Sweden
AV154	87639	oat	Enbaku	*Avena sativa*	South Korea
AV155	269146	oat	Maldwyn HF/59	*Avena sativa*	United Kingdom
AV156	87641	oat	Enbaku	*Avena sativa*	South Korea
AV157	269148	oat	Opus	*Avena sativa*	Denmark
AV158	103672	oat	Advocate	*Avena sativa*	Australia
AV159	269170	oat	936	*Avena sativa*	Turkey
AV16	232886	oat	Szekacs 8	*Avena sativa*	Hungary
AV160	156016	oat	Shatilovskij 56	*Avena sativa*	Georgia
AV161	269171	oat	948	*Avena sativa*	Turkey
AV162	CIav732	oat	Banner	*Avena sativa*	USA
AV163	269172	oat	4285	*Avena sativa*	Turkey
AV164	CIav751	oat	Banner	*Avena sativa*	USA
AV165	269173	oat	4286	*Avena sativa*	Turkey
AV166	CIav754	oat	Early Mountain	*Avena sativa*	Turkey
AV167	269174	oat	4287	*Avena sativa*	Turkey
AV168	CIav804	oat	Sparrowbill	*Avena sativa*	New Zealand
AV169	269176	oat	4290	*Avena sativa*	Turkey
AV170	CIav831	oat	Aurora	*Avena sativa*	USA
AV171	CIav1794	oat	Gray	*Avena sativa*	USA
AV172	CIav9323	oat	Pennsylvania 31	*Avena sativa*	USA
AV173	CIav1995	oat	Kilby Hull-less	*Avena sativa*	China
AV174	CIav9324	oat	Pennsylvania 41	*Avena sativa*	USA
AV175	CIav2783	oat	Sandy	*Avena sativa*	United Kingdom
AV176	CIav9325	oat	Pennsylvania 52	*Avena sativa*	USA
AV177	CIav2829	oat	Dookie 10	*Avena sativa*	Australia
AV178	CIav9337	oat	Pennsylvania 72	*Avena sativa*	USA
AV179	CIav4744	oat	S 175	*Avena sativa*	United Kingdom
AV18	235278	oat	16129	*Avena sativa*	France
AV180	CIav9341	oat	Pennsylvania 72	*Avena sativa*	USA
AV181	CIav5043	oat	S-175	*Avena sativa*	United Kingdom
AV182	CIav9347	oat	Pennsylvania 82	*Avena sativa*	USA
AV183	CIav6493	oat	Cl 6493	*Avena sativa*	USA
AV184	CIav9423	oat	Rodney PGA	*Avena sativa*	USA
AV185	CIav8099	oat	833-Gu63	*Avena sativa*	Australia
AV186	40650	oat	Chinese Hull-less	*Avena sativa*	China
AV187	CIav8123	oat	Kentucky 63-885	*Avena sativa*	USA
AV188	40651	oat	2185a	*Avena sativa*	China
AV189	CIav8126	oat	Pennsylvania 59	*Avena sativa*	USA
AV190	50351	oat	Golden Yellow	*Avena sativa*	Germany

AV191	CIav8207	oat	Kentucky 64-967	*Avena sativa*	USA
AV192	174576	oat	Rougette d'Esta	*Avena sativa*	France
AV193	CIav8251	oat	Kyto	*Avena sativa*	Finland
AV194	174585	oat	Panache de Roy	*Avena sativa*	France
AV195	CIav8440	oat	Corvallis 59-186	*Avena sativa*	USA
AV196	175524	oat	Esa	*Avena sativa*	Finland
AV197	CIav8442	oat	Nodaway 70	*Avena sativa*	USA
AV198	175526	oat	Simo	*Avena sativa*	Finland
AV199	CIav9082	oat	Red Algerian Sel	*Avena sativa*	Algeria
AV2	264260	Oat	Lightning	*Avena sativa*	Sweden
AV20	251900	oat	Shatilovskij 56	*Avena sativa*	Georgia
AV200	180913	oat	Borriesa	*Avena sativa*	Germany
AV201	184012	oat	207	*Avena sativa*	Serbia
AV202	158220	oat	Myeaj	*Avena sativa*	Georgia
AV203	184015	oat	210	*Avena sativa*	Serbia
AV204	158221	oat	WIR 4073/44	*Avena sativa*	Mongolia
AV205	184017	oat	212	*Avena sativa*	Serbia
AV206	174504	oat	Avoine de Pirnija	*Avena sativa*	France
AV207	184018	oat	213	*Avena sativa*	Serbia
AV208	158240	oat	Mutica	*Avena sativa*	Bosnia and Herzegovina
AV209	184019	oat	214	*Avena sativa*	Bosnia and Herzegovina
AV210	174551	oat	Unilaterale Hativ	*Avena sativa*	France
AV211	184022	oat	217	*Avena sativa*	Croatia
AV212	CIav1694	oat	White Maine	*Avena sativa*	USA
AV213	184026	oat	221	*Avena sativa*	Serbia
AV214	CIav1714	oat	White Queen	*Avena sativa*	USA
AV215	184028	oat	223	*Avena sativa*	Serbia
AV216	CIav1773	oat	Praegravis Scoti	*Avena sativa*	USA
AV217	184033	oat	228	*Avena sativa*	Serbia
AV218	CIav1774	oat	Praegravis Gerrn	*Avena sativa*	USA
AV219	193027	oat	Scotland Club	*Avena sativa*	United Kingdom
AV22	197651	oat	Bambu II	*Avena sativa*	Sweden
AV220	CIav1777	oat	Tabor	*Avena sativa*	Czech Republic
AV24	197835	oat	Beiar	*Avena sativa*	Norway
AV26	199677	oat	Golden Rain	*Avena sativa*	Sweden
AV28	220870	oat	Dale	*Avena sativa*	Australia
AV30	221279	oat	Esa	*Avena sativa*	Finland
AV31	436093	oat	393	*Avena sativa*	Chile
AV32	CIav1466	oat	White Tartar	*Avena sativa*	USA
AV33	436102	oat	515	*Avena sativa*	Chile
AV34	CIav1564	oat	Eclipse	*Avena sativa*	USA
AV36	CIav1567	oat	Sensation	*Avena sativa*	USA
AV38	CIav1572	oat	Black Alaska	*Avena sativa*	USA
AV4	264261	oat	Golden Rain II	*Avena sativa*	Sweden
AV40	CIav1574	oat	Boswell`s Black	*Avena sativa*	USA
AV41	258638	oat	Jogeva Roste Kip	*Avena sativa*	Estonia
AV42	CIav1576	oat	Victory	*Avena sativa*	Sweden
AV43	258670	oat	*none*	*Avena sativa*	Georgia
AV44	CIav1579	oat	Cl 1579	*Avena sativa*	South Africa
AV45	258699	oat	Khibiny 1	*Avena sativa*	Georgia

AV46	CIav1582	oat	Silvermine	*Avena sativa*	USA
AV47	260891	oat	Picton	*Avena sativa*	United Kingdom
AV48	CIav1588	oat	College Success	*Avena sativa*	USA
AV49	264259	oat	Nip	*Avena sativa*	Sweden
AV50	CIav1589	oat	College Wonder	*Avena sativa*	USA
AV51	180931	oat	Kraftts Rhein	*Avena sativa*	Germany
AV52	269177	oat	4291	*Avena sativa*	Turkey
AV53	180948	oat	Wadsacks Gelb	*Avena sativa*	Germany
AV55	180949	oat	Wentzels Ander	*Avena sativa*	Germany
AV56	269180	oat	4294	*Avena sativa*	Turkey
AV57	182490	oat	Kyto	*Avena sativa*	Finland
AV58	269869	oat	808	*Avena sativa*	Pakistan
AV59	182492	oat	Golden Rain	*Avena sativa*	Sweden
AV6	264268	oat	Great Mogul II	*Avena sativa*	Sweden
AV60	273868	oat	2028	*Avena sativa*	Ethiopia
AV61	287279	oat	AVE 274/62	*Avena sativa*	Albania
AV62	CIav1639	oat	White Bonanza	*Avena sativa*	USA
AV63	287394	oat	Badberger	*Avena sativa*	Germany
AV64	Clav1651	oat	White Maine	*Avena sativa*	USA
AV65	287406	oat	Pin Lan Che U Sac	*Avena sativa*	China
AV66	CIav1656	oat	Probsteier	*Avena sativa*	Sweden
AV67	287462	oat	AVE 288/59	*Avena sativa*	Greece
AV68	CIav1658	oat	Romana	*Avena sativa*	USA
AV69	290029	oat	Kompolti	*Avena sativa*	Hungary
AV70	CIav1673	oat	Wernich Golden	*Avena sativa*	USA
AV71	294673	oat	Jezewski	*Avena sativa*	Poland
AV73	294676	oat	Mlochowski	*Avena sativa*	Poland
AV75	294687	oat	Mongolia	*Avena sativa*	Bulgaria
AV77	296147	oat	Verhniacskij 53	*Avena sativa*	United Kingdom
AV79	296163	oat	WIR 3960	*Avena sativa*	United Kingdom
AV8	264744	oat	Hokuyo	*Avena sativa*	Japan
AV81	266278	oat	WIR 3969	*Avena sativa*	Georgia
AV83	266280	oat	WIR 6103	*Avena sativa*	Georgia
AV85	266284	oat	WIR 9523	*Avena sativa*	Belarus
AV86	447272	oat	A-0146	*Avena sativa*	China
AV87	266288	oat	WIR 10780	*Avena sativa*	United Kingdom
AV88	447278	oat	Harman	*Avena sativa*	China
AV89	266289	oat	L'govskij 179	*Avena sativa*	Georgia
AV90	447296	oat	Xin Yuan	*Avena sativa*	China
AV91	266830	oat	62	*Avena sativa*	United Kingdom
AV92	264856	oat	830	*Avena sativa*	Serbia
AV93	266857	oat	Dunn's	*Avena sativa*	New Zealand
AV94	264961	oat	1053	*Avena sativa*	Croatia
AV95	267074	oat	Stepiak 648	*Avena sativa*	Georgia
AV96	265454	oat	Eho	*Avena sativa*	Finland
AV97	267078	oat	Jogeva Hamarik	*Avena sativa*	Estonia
AV98	265455	oat	Gliden Rain II	*Avena sativa*	Sweden
AV99	267079	oat	L'govskij 1026	*Avena sativa*	Georgia

Table S2 Descriptive statistics of seven agronomic traits in multiple environments

trait	loci	min	max	mean	standard deviation	efficient of Variation (%)
TN	2022HY	10.20	92.20	39.86	14.60	0.37
	2022CD	6.33	38.50	14.76	4.66	0.32
	2023HY	10.20	72.60	35.86	10.97	0.31
	2023CD	5.67	53.00	20.35	8.49	0.42
SD	2022HY	2.72	10.26	7.03	1.31	0.19
	2022CD	3.82	9.58	6.80	0.96	0.14
	2023HY	3.11	10.80	7.22	1.22	0.17
	2023CD	3.64	9.54	6.47	1.15	0.18
FLL	2022HY	9.18	48.26	24.49	5.75	0.23
	2022CD	6.86	46.80	26.70	5.92	0.22
	2023HY	12.70	44.98	25.98	5.91	0.23
	2023CD	9.44	39.24	24.61	6.01	0.24
FLW	2022HY	0.72	3.58	2.20	0.53	0.24
	2022CD	1.60	4.16	2.85	0.52	0.18
	2023HY	0.86	6.54	2.61	0.69	0.26
	2023CD	1.42	6.96	2.51	0.58	0.23
CL	2022HY	14.38	60.88	38.84	9.05	0.23
	2022CD	24.56	83.50	46.19	10.74	0.23
	2023HY	21.62	54.96	39.22	7.17	0.18
	2023CD	22.44	58.72	41.21	7.22	0.18
SPP	2022HY	11.80	247.80	95.23	45.43	0.48
	2022CD	23.67	188.40	103.06	32.50	0.32
	2023HY	14.52	198.72	91.65	41.25	0.44
	2023CD	38.40	304.40	133.43	46.29	0.35
PH	2022HY	58.40	169.40	118.64	15.79	0.13
	2022CD	90.40	160.00	128.47	15.12	0.12
	2023HY	80.96	173.40	127.36	16.39	0.13
	2023CD	103.92	202.16	160.85	16.79	0.10

PH, represent plant height; SD, represent stem diameter; FLL and FLW represent flag leaf length and flag leaf width, respectively; CL, represent cob length; TN, represent tiller number; SPP, represent spikelets per panicle.

Table S3 Heritability of seven agronomic traits

	FLW	FLL	TN	SPP	PH	SD	CL
heritability	0.58	0.59	0.71	0.55	0.72	0.76	0.65

PH, represent plant height; SD, represent stem diameter; FLL and FLW represent flag leaf length and flag leaf width, respectively; CL, represent cob length; TN, represent tiller number; SPP, represent spikelets per panicle.

Table S4. Best model selection for GWAS for 7 agronomic traits

	2022HY	2022CD	2023HY	2023CD	BLUP
TN	BLINK	SUPER	BLINK	SUPER	BLINK
PH	BLINK	FarmCPU	BLINK	SUPER	FarmCPU
FLW	EMMMAX	FarmCPU	EMMMAX	EMMMAX	BLINK
FLL	EMMMAX	BLINK	EMMMAX	BLINK	BLINK
SPP	FarmCPU	BLINK	BLINK	BLINK	BLINK
CL	FarmCPU	EMMMAX	BLINK	SUPER	BLINK
SD	FarmCPU	EMMMAX	BLINK	BLINK	BLINK

PH, represent plant height; SD, represent stem diameter; FLL and FLW represent flag leaf length and flag leaf width, respectively; CL, represent cob length; TN, represent tiller number; SPP, represent spikelets per panicle.

Table S5. Important SNP markers associated with seven different agronomic traits in oat.

agronomic trait	envs_information	taxa	chr	Position	P.value	maf	effect
TN	2022_HY	chr1C:54169184	chr1C	54169184	1.02187E-10	0.0631579	17.79205081
		chr2D:25512681	chr2D	25512681	1.53463E-10	0.0868421	9.121942791
		chr3D:422895772	chr3D	422895772	9.52001E-08	0.0973684	-11.23987027
		chr4D:404763300	chr4D	404763300	1.3702E-07	0.1763158	-4.253261671
	2023_HY	chr7C:54538195	chr7C	54538195	1.13693E-12	0.0316901	-18.73886022
		chr3D:379166305	chr3D	379166305	4.85375E-08	0.0457746	11.84514164
		chr5D:418129359	chr5D	418129359	1.79622E-07	0.3838028	3.586769271
	BLUP	chr6C:37537450	chr6C	37537450	3.98541E-07	0.2111	2.621621875
PH	2022_HY	chr5C:106487816	chr5C	106487816	9.26026E-10	0.0625	7.16546413
		chr7D:467618105	chr7D	467618105	1.0419E-08	0.0896739	-8.42841316
		chr2C:490869189	chr2C	490869189	7.89147E-08	0.1141304	-6.858946465
		chr3D:284563	chr3D	284563	4.44073E-07	0.1766304	4.594546173
		chr5D:65901066	chr5D	65901066	5.15413E-07	0.0652174	-7.126496847
	2022_CD	chr7A:237848033	chr7A	237848033	6.83816E-11	0.265625	-7.303547346
		chr6C:552275705	chr6C	552275705	2.20372E-10	0.4166667	6.847674865
		chr6A:103519117	chr6A	103519117	1.81798E-08	0.1614583	-12.78989596
		chr1D:357525849	chr1D	357525849	7.08801E-08	0.1041667	8.825576617
		chr6C:89199800	chr6C	89199800	2.95174E-07	0.078125	-6.80673103
		chr7C:338655578	chr7C	338655578	5.88651E-07	0.1822917	3.919643785
	2023_CD	chr7C:43068188	chr7C	43068188	1.07127E-07	0.0486111	-16.79066845
		chr5D:486284059	chr5D	486284059	4.36385E-07	0.0659722	-30.76748107
		chr5D:486283973	chr5D	486283973	4.36385E-07	0.0659722	30.76748107
		chr5C:527842146	chr5C	527842146	5.67728E-07	0.0694444	-18.19723372
		chr7C:43058610	chr7C	43058610	5.93753E-07	0.0590278	13.65176714
	BLUP	chr5C:143294695	chr5C	143294695	5.48471E-10	0.0520833	-3.896410087
		chr5A:40174605	chr5A	40174605	3.98183E-09	0.0885417	2.471048128
		chr1D:10442407	chr1D	10442407	2.60907E-08	0.0625	-4.97895796
		chr7C:41902804	chr7C	41902804	2.59953E-07	0.1171875	-0.696326261
	2022_HY	chr6A:270094076	chr6A	270094076	5.01238E-08	0.0706522	25.58272337
		chr4C:618540534	chr4C	618540534	1.28056E-07	0.1657609	17.328922

		chr1A:259838680	chr1A	259838680	2.33267E-07	0.1032609	22.04909042
		chr4C:588104097	chr4C	588104097	1.17402E-06	0.1467391	16.4488056
		chr6C:69973159	chr6C	69973159	4.07489E-11	0.2730769	21.54646413
SPP	2022_CD	chr1A:311739654	chr1A	311739654	8.22672E-11	0.3923077	14.45849668
		chr5C:480683327	chr5C	480683327	9.15277E-09	0.2230769	-17.1769799
		chr7A:253010786	chr7A	253010786	9.74E-10	0.2336957	20.86776222
	2023_HY	chr6A:315098362	chr6A	315098362	3.50E-07	0.4918478	-41.85405816
		chr6A:341302474	chr6A	341302474	8.72E-07	0.2961957	19.22657538
	BLUP	chr2C:6893316	chr2C	6893316	1.64E-07	0.15625	-14.13809318
		chr7C:231276974	chr7C	231276974	4.59E-07	0.0546875	-49.84875751
		chr2D:24123346	chr2D	24123346	3.99675E-10	0.1277174	4.047461917
	2022_HY	chr7D:477775749	chr7D	477775749	1.75778E-07	0.111413	-5.402187923
		chr6D:149990323	chr6D	149990323	6.10089E-07	0.1521739	-4.453151924
		chr7D:470270105	chr7D	470270105	1.43936E-14	0.1126761	-9.4789282
CL		chr2C:146851079	chr2C	146851079	1.01893E-07	0.0246479	-13.79023414
	2023_HY	chr4A:355454128	chr4A	355454128	1.1641E-07	0.1302817	-3.473221234
		chr1D:338951348	chr1D	338951348	1.27009E-06	0.1408451	3.241032729
		chr1D:335505167	chr1D	335505167	2.32841E-06	0.1760563	2.958085145
	BLUP	chr2C:203544127	chr2C	203544127	1.97797E-07	0.0963542	-7.813390041
		chr1A:77482269	chr1A	77482269	2.36091E-09	0.0706522	0.812651714
	2022_HY	chr3D:440551973	chr3D	440551973	1.72072E-07	0.3586957	0.130434783
		chr3C:533121749	chr3C	533121749	3.96695E-07	0.0842391	0.684794914
SD		chr2C:494637843	chr2C	494637843	1.05755E-09	0.1028369	-0.419743676
	2023_CD	chr5C:98544403	chr5C	98544403	5.89554E-09	0.1170213	0.505210482
		chr1C:347499288	chr1C	347499288	3.07983E-07	0.0602837	-0.44393573
		chr6C:45013901	chr6C	45013901	4.55818E-07	0.0780142	-0.528902189
	BLUP	chr7D:376592511	chr7D	376592511	2.00214E-07	0.078125	0.855468435
		chr3C:549420928	chr3C	549420928	2.61253E-10	0.4928571	-0.26353102
	2022_CD	chr5D:19282280	chr5D	19282280	9.13757E-08	0.1214286	0.257241849
		chr1D:273185279	chr1D	273185279	2.19482E-07	0.0928571	-0.222728668
		chr6C:564717336	chr6C	564717336	6.54137E-07	0.0821429	0.436763297
		chr3A:378078108	chr3A	378078108	2.18744E-14	0.4964789	-2.872177051

FLW	2023_HY	chr6C:574691341	chr6C	574691341	4.18963E-11	0.0457746	3.648232513
		chr6C:575190923	chr6C	575190923	1.61539E-08	0.0528169	-2.515504781
		chr6C:25741715	chr6C	25741715	3.76306E-08	0.0669014	-2.687185374
		chr6C:574790895	chr6C	574790895	1.33344E-07	0.0739437	2.224339222
		chr4D:218192187	chr4D	218192187	5.62901E-07	0.0387324	-4.034104491
		chr4D:218338073	chr4D	218338073	5.62901E-07	0.0387324	-4.034104491
		chr6C:25868029	chr6C	25868029	6.3091E-07	0.0457746	2.845002537
		chr2A:194537978	chr2A	194537978	6.43734E-07	0.0669014	2.892699163
	BLUP	chr3A:378078108	chr3A	378078108	7.64E-36	0.4973958	-0.730008189
		chr2C:478893988	chr2C	478893988	3.055E-13	0.1927083	0.058900275
		chr5C:110343607	chr5C	110343607	6.93181E-10	0.1067708	-0.046623317
		chr4D:389477752	chr4D	389477752	1.52732E-08	0.0859375	-0.049405474
		chr1D:326632727	chr1D	326632727	3.04306E-08	0.2369792	-0.284984168
		chr6C:25741715	chr6C	25741715	3.14455E-08	0.1171875	-0.063227012
		chr5C:550963036	chr5C	550963036	7.53352E-07	0.1223958	-0.041915897
	2022_HY	chr5C:541689226	chr5C	541689226	8.57071E-07	0.0651042	0.200520833
		chr4C:625934823	chr4C	625934823	2.61512E-06	0.0706522	5.405501466
		chr6A:315098362	chr6A	315098362	3.61E-06	0.4918478	-6.011648285
	2022_CD	chr5C:416214721	chr5C	416214721	5.25724E-08	0.0931034	-4.776344578
		chr7C:360132428	chr7C	360132428	1.13706E-05	0.1758621	3.191002092
FLL	2023_HY	chr2C:381495777	chr2C	381495777	1.32178E-11	0.0457746	104.2763666
		chr2C:379031522	chr2C	379031522	2.62124E-09	0.0598592	81.29389217
		chr3D:247283611	chr3D	247283611	2.3494E-08	0.4929577	-139.5386784
		chr4D:331031904	chr4D	331031904	2.19171E-07	0.0528169	66.38355185
		chr3D:247283245	chr3D	247283245	2.56326E-07	0.4894366	114.9180231
		chr3D:58665799	chr3D	58665799	3.15688E-07	0.0633803	60.172655
		chr3C:42403143	chr3C	42403143	3.64849E-07	0.0316901	104.7706702
		chr2C:381478674	chr2C	381478674	8.34938E-07	0.0739437	-52.04719617
	2023_CD	chr4D:277799160	chr4D	277799160	3.0173E-10	0.1597222	3.492515892
		chr5A:16429536	chr5A	16429536	1.73922E-07	0.2361111	2.694644857
		chr5A:16209941	chr5A	16209941	2.78331E-07	0.1701389	2.787100531
	BLUP	chr5A:16429536	chr5A	16429536	1.46723E-06	0.2486911	0.636445667

PH, represent plant height; SD, represent stem diameter; FLL and FLW represent flag leaf length and flag leaf width, respectively; CL, represent cob length; TN, represent tiller number; SPP, represent spikelets per panicle.

Table S6. Mean Square Error (MSE) and Mean Absolute Error (MAE) of the eight methods were evaluated in 500 replicates at 5-fold CVs

Method	FLW		FLL		TN		SN		SD		PH		CL	
	MSE	MAE	MSE	MAE	MSE	MAE	MSE	MAE	MSE	MAE	MSE	MAE	MSE	MAE
ElasticNet	0.0078	0.0001	0.111	0.012	17.903	320.53	14.859	220.78	0.109	0.012	15.760	248.36	12.981	168.50
Lasso	0.0084	0.0001	0.114	0.013	17.534	307.43	14.695	215.94	0.108	0.012	15.545	241.65	13.058	170.51
Ridge	0.0080	0.0001	0.112	0.012	17.761	315.47	14.730	216.96	0.107	0.011	15.564	242.24	13.107	171.79
KRR	0.0079	0.0001	0.120	0.014	17.656	311.74	14.341	205.68	0.107	0.011	15.162	229.88	12.965	168.08
SVR-linear	0.0096	0.0001	0.115	0.013	17.957	322.46	14.660	214.92	0.105	0.011	15.207	231.26	13.129	172.37
SVR-poly	0.0090	0.0001	0.116	0.013	17.311	299.66	15.519	240.85	0.106	0.011	15.665	245.40	13.469	181.42
GBLUP	0.0102	0.0001	0.133	0.018	19.206	368.87	15.756	248.26	0.109	0.012	16.815	282.75	13.373	178.84

PH, represent plant height; SD, represent stem diameter; FLL and FLW represent flag leaf length and flag leaf width, respectively; CL, represent cob length; TN, represent tiller number; SPP, represent spikelets per panicle.
